# The patellofemoral morphology and the normal predicted value of tibial tuberosity-trochlear groove distance in the Chinese population

**DOI:** 10.1186/s12891-021-04454-8

**Published:** 2021-06-23

**Authors:** Zhe Li, Guanzhi Liu, Run Tian, Ning Kong, Yue Li, Yiyang Li, Kunzheng Wang, Pei Yang

**Affiliations:** Department of Bone and Joint Surgery, The Second Affiliated Hospital of Medical College, Xi’an Jiaotong University, Shaanxi 710004 Xi’an, People’s Republic of China

**Keywords:** Patellofemoral joint, Tibial tuberosity-trochlear groove distance, Knee morphology, LASSO regression

## Abstract

**Background:**

Our objective was to obtain normal patellofemoral measurements to analyse sex and individual differences. In addition, the absolute values and indices of tibial tuberosity-trochlear groove (TT-TG) distances are still controversial in clinical application. A better method to enable precise prediction is still needed.

**Methods:**

Seventy-eight knees of 78 participants without knee pathologies were included in this cross-sectional study. A CT scan was conducted for all participants and three-dimensional knee models were constructed using Mimics and SolidWorks software. We measured and analysed 19 parameters including the TT-TG distance and dimensions and shapes of the patella, femur, tibia, and trochlea. LASSO regression was used to predict the normal TT-TG distances.

**Results:**

The dimensional parameters, TT-TG distance, and femoral aspect ratio of the men were significantly larger than those of women (all *p* values < 0.05). However, after controlling for the bias from age, height, and weight, there were no significant differences in TT-TG distances and anterior-posterior dimensions between the sexes (all *p* values > 0.05). The Pearson correlation coefficients between the anterior femoral offset and other indexes were consistently below 0.3, indicating no relationship or a weak relationship. Similar results were observed for the sulcus angle and the Wiberg index. Using LASSO regression, we obtained four parameters to predict the TT-TG distance (R^2^ = 0.5612, *p* < 0.01) to achieve the optimal accuracy and convenience.

**Conclusions:**

Normative data of patellofemoral morphology were provided for the Chinese population. The anterior-posterior dimensions of the women were thicker than those of men for the same medial-lateral dimensions. More attention should be paid to not only sex differences but also individual differences, especially the anterior condyle and trochlea. In addition, this study provided a new method to predict TT-TG distances accurately.

**Supplementary Information:**

The online version contains supplementary material available at 10.1186/s12891-021-04454-8.

## Background

Although total knee arthroplasty (TKA) has proven to be a successful surgical procedure for alleviating pain and improving function in patients with knee osteoarthritis, patient satisfaction rates after TKA vary between 75 and 89 % [1]. Anterior knee pain is a major reason for dissatisfaction, which may be caused by a variety of abnormalities, including patellofemoral pathologies [2]. An increasing number of researchers have observed mismatches of the patellofemoral joints after TKA. Matz et al. reported that the probabilities of changes in anterior femoral offset, anteroposterior size of the femur and anterior patellar offset after TKA were 40 %, 60 %, and 71 %, respectively, compared with those before TKA [3]. Kalichman et al. suggested that increased trochlear angles were associated with exacerbated functional impairments [4]. Moreover, Jan et al. highlighted that the patellofemoral geometry was of great importance in TKA, but was often overlooked [5]. Thus, increasing attention has been devoted to the modified design of patellofemoral joint prostheses in the field of TKA [6], among which the study of patellofemoral morphology is the basic research focus.

In the last few decades, researchers have reported the shape differences of knees between ethnicity and sex where there are relatively few studies on the patellofemoral joint. An extensive study from Mahfouz et al. analysed 1000 normal adult knees to identify differences in three-dimensional knee morphology among white American, African American, and East Asian populations, calculating 11 femoral and 9 tibial measurements [7]. Asseln et al. comprehensively analysed 412 pathological knees following TKA using 33 femoral and 21 tibial features to investigate sex differences and they indicated that large interindividual variations should also be important for specific implant design despite sex differences [8]. Yue et al. investigated the morphologic measurements of the femur and tibia in healthy Chinese and white participants. They found that Chinese women have a narrower distal femur and described the differences in knee anthropometry between sexes and ethnicities [9]. Although most of the literature affirmed sex differences between knee morphologies, several studies suggested no sex differences regarding anterior and posterior condylar of the distal femur [10, 11]. Additionally, relatively few studies on sex differences in patellofemoral morphology have been investigated, and most of them analysed the patella but not the anterior and posterior condylar regions of the femur or entire patellofemoral joint [12–14]. Thus, the sexual dimorphism of the entire patellofemoral morphology is still unclear. However, many studies have shown that anatomical differences of the knees, especially the distal femurs, are not only sex differences but also individual differences [8, 15–17]. Taken together, studies on the sex differences and individual differences of the entire patellofemoral morphology are still needed.

The tibial tubercle-trochlear groove (TT-TG) distance is a well-established reliable index that evaluates tibial tubercle lateralization and patellofemoral instability [18, 19]. A TT-TG value of > 15 mm is recommended as abnormal, and a value of > 20 mm is the threshold for performing tibial tubercle osteotomy [20, 21]. However, this absolute value does not account for the anatomic differences between sex and ethnicity, and it was hypothesized that the TT-TG distance is highly correlated with knee size [22–24]. An increasing number of researchers have raised disputes about inaccurate absolute value results [25, 26]. Thus, Cao et al. described the application of the TT-TG indices (ratio of the TT-TG distance to the tibial maximal mediolateral axis), and the result needed to be further confirmed [27]. Hernigou et al. predicted the normal TT-TG distances in Belgium using the femoral width and tibial width for the first time. However, the mediolateral width of the femur and tibia might not be the best parameters to describe the knee sizes, and similar parameters also included the height and weight of the patient [28]. Thus, it is still necessary to analyse appropriate parameters to predict the normal TT-TG distance and to create the reference criterion in Chinese individuals.

Therefore, we hypothesized that (a) most parameters of entire patellofemoral morphology including the dimensions of the patellofemoral joints and the shape of the distal femur exhibit sexual dimorphism except the anterior and posterior condylar of the distal femur; (b) patellofemoral morphology exhibits individual differences; and (c) the TT-TG distance is moderately correlated with knee size. By screening relevant parameters, the TT-TG distance can be predicted by more complex and accurate methods compared with the current methods proposed in the above literature.

## Methods

### Participant demographics

Seventy-eight participants (38 women) were recruited from the neighbouring communities of the Second Affiliated Hospital of Xi’an Jiaotong University from May 2017 to October 2017. The local ethics committee of the hospital approved the project. Informed consent was obtained from all included participants. Only one side of the knee was chosen to be studied and the left or right knee was chosen at random to maintain the independence of the data.

### Inclusion and exclusion criteria

The inclusion criteria were as follows: (1) age ≥ 18 years; (2) height greater than 155 cm and less than 190 cm; (3) only asymptomatic and nonpathological joints were included in this research, which were verified through clinical examination and CT images.

The exclusion criteria were as follows: (1) pregnant women or those who plan to conceive within the next year; (2) history of poliomyelitis, rickets, dwarfism, rheumatoid arthritis, or other diseases that affect lower limbs; (3) a congenital deformity in either lower limb; (4) history of patellar instability, patellar dislocation, or the patellar apprehension test was positive on physical examination; (5) osteoarthritis of either hip or knee confirmed by previous imaging or by current CT scan; (6) fracture caused by injury to femur, tibia, hip, or knee; (7) joint arthroplasty, arthroscopic surgery or other surgery on the hip or knee.

### CT Protocol and creation of 3-dimensional knee model

A CT scan of the lower limb was obtained using a helical CT scanner (120 kV, 200 mA, reconstruction thickness 0.6 mm, reconstruction spacing 0.4 mm, GE revolution CT, General Electric Company, Milwaukee, Wis). All participants were supine and non-weightbearing as per the protocol with the extension of all knees. All DICOM images were imported and segmented in Mimics software (version 17.0, Materialise Inc., Leuven, Belgium), which exported the 3-dimensional reconstructions of the tibia, femur, and patella. The reconstructed models were then processed with SolidWorks engineering software (version 2017, Dassault Systemes Company, Concord, Massachusetts). Using SolidWorks software, we adjusted the position of the models, obtained the standard radiographic views, constructed appropriate coordinate systems, and finally transformed the 3D models to planar images to finish the measurements accurately. Six standard radiographic views, including lower and lateral views of the patella and femur, axial views simulated in 30° knee flexion of the femur, and upper view of the tibia, were obtained. We defined the lower view of the patella as the view observed from the lower pole of the patella with the central ridge of the patella completely upward. The lateral view of the patella was defined as the view observed from the side of the patella with the central ridge of the patella completely rightward. We defined the lower view of the femur as the view observed below the distal femur with maximum mediolateral and anteroposterior sizes of the femur obtained. The lateral view of the femur was defined as the view observed from the side of the femur with medial and lateral condyles overlapping completely. We defined the upper view of the tibia as the view observed above the tibia with maximum mediolateral and anteroposterior sizes of the tibia obtained. In addition, we adjusted the position of the femur and angle of our observation to mimic the axial views simulated in 30° knee flexion of the femur. The specific operation was as follows: we first placed the femur in the lateral view, rotated the femur along the axis perpendicular to the lateral view so that the long axis of the femur formed an angle of 30° with the vertical line, then established the coordinate system, switched the femur to the lower view, and finally mimicked the axial views of the femur with 30° knee flexion.

### Definitions and measurements of parameters

**Fig. 1:**
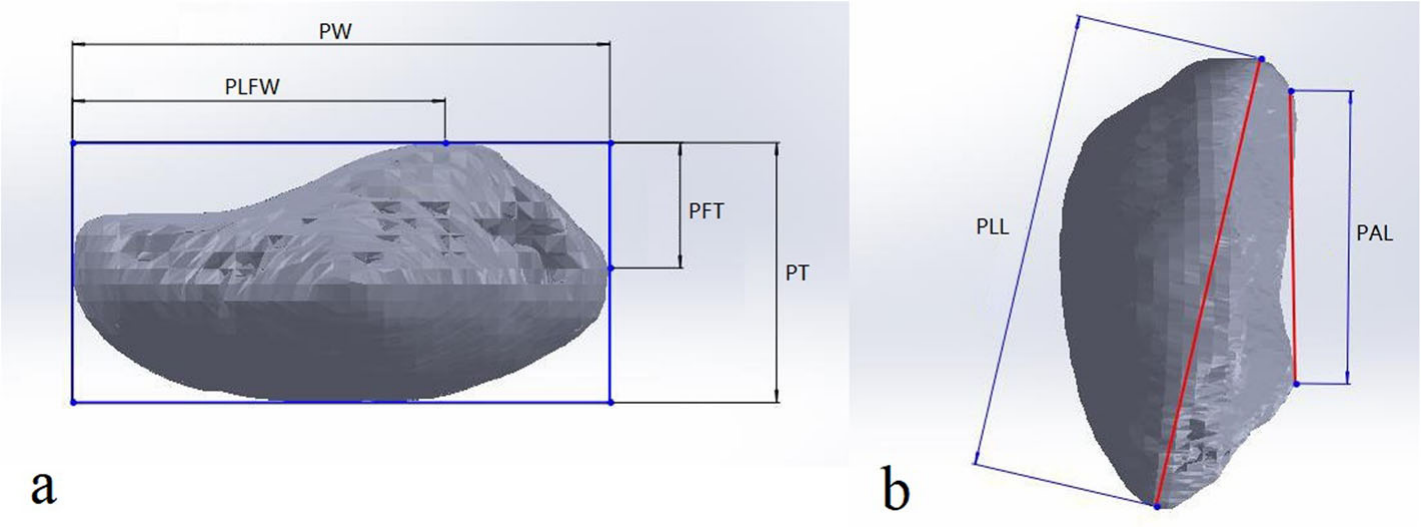
**a** Patella thickness (PT), patella facet thickness (PFT), patella width (PW), patella lateral facet width (PLFW); **b** longitudinal length of the whole patella (PLL) and longitudinal length of the articulating surface of the patella (PAL)

In the lower view of the patella, we measured the four following parameters as described by Muhamed et al. [13] (Fig. 1a):

1. Patella width (PW): the distance between the tangent line of the medial margin and the tangent line of the lateral margin of the patella.
2. Patella lateral facet width (PLFW): the distance between the most prominent point of the central ridge and the tangent line of the lateral margin of the patella.
3. Patella thickness (PT): the distance between the most prominent point of the central ridge and the tangent line of the anterior margin of the patella.
4. Patella facet thickness (PFT): the distance between the most prominent point of the central ridge and the tangent line of the deepest margin of the patella facet.

Additionally, the Wiberg index (PLFW/PW) was calculated [29], and the morphology of the patella was determined by the Wiberg classification as modified by Baumgartl and Ficat [30].

In the lateral view of the patella, we measured the two following parameters described by Yoo et al. [31] (Fig. 1b):

1. Longitudinal length of the whole patella (PLL): the distance between the most prominent point in the upper pole and the most prominent point in the lower pole of the patella.
2. Longitudinal length of the articular surface of the patella (PAL): the distance between the upper margin and the lower margin of the articular surface of the patella.

**Fig. 2:**
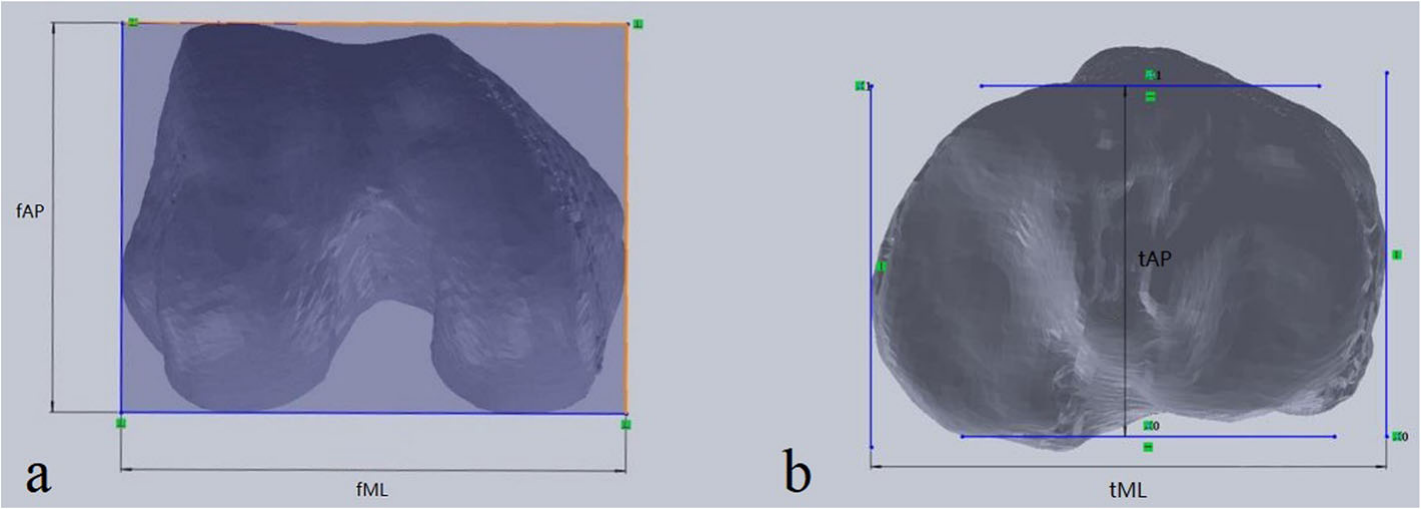
**a** The mediolateral (fML) and anteroposterior (fAP) sizes of the femur; **b** the mediolateral (tML) and anteroposterior (tAP) sizes of the tibia

In the lower view of the femur, we measured the two following parameters as described by Yue et al. [9] (Fig. 2a):

The mediolateral (fML) and anteroposterior (fAP) sizes of the femur: taking the posterior condylar line (PCL, the line along the most posterior margins on each condyle) as a reference, a rectangular bounding box that fitted the distal femur was created. The fML and fAP values were measured using the bounding box. Additionally, the femoral aspect ratio (fML/fAP) was calculated.

In the upper view of the tibia, we measured the two following parameters as described by Mahfouz et al. [7] (Fig. 2b):

1. The mediolateral width of the tibia (tML): the maximum width of the tibial plateau in the mediolateral direction.
2. The anteroposterior size of the tibia (tAP): the maximum length of the tibial plateau in the anteroposterior direction, through the midpoint of the intercondylar eminence.

**Fig. 3:**
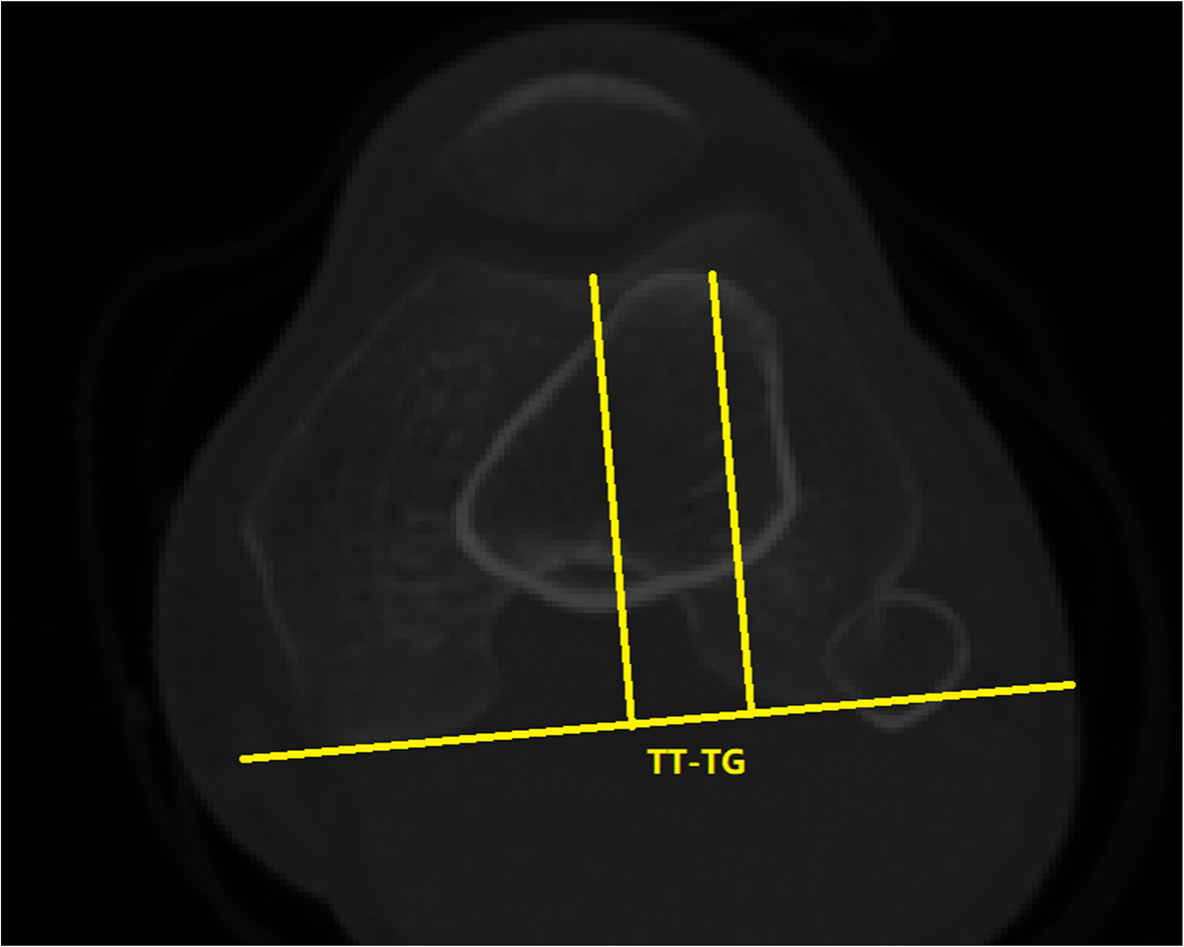
TT-TG distance

We measured the TT-TG distance as described by Hernigou et al. [28] The TT-TG distance was defined as the distance between the most anterior point of the tibial tuberosity and the deepest point of the trochlear groove, parallel to and reference to the PCL (Fig. 3). CT images were observed in Mimics software and the transverse picture of the most anterior point of the tibial tuberosity was first selected. Next, the transverse picture of the proximal trochlea at the level of the “roman arch” was selected. The two pictures were processed and merged using ImageJ software (the National Institutes of Health, Bethesda, MD, USA) and the TT-TG distance was measured in the final merged picture. All transverse pictures were kept perpendicular to the vertical axis of the lower limbs, implemented through online reslice of Mimics software, to maintain the accuracy and consistency of the obtained data. The specific operation was as follows: we first determined the femoral mechanical axis as described by previous studies [32, 33] and obtained a new axis by rotating the mechanical axis inwards by 3° on the coronal plane, which was defined as the vertical axis. Then, we resliced the CT images along the vertical axis to obtain the transverse images perpendicular to the vertical axis. Finally, we measured the TT-TG distances accurately using the new transverse images.

**Fig. 4:**
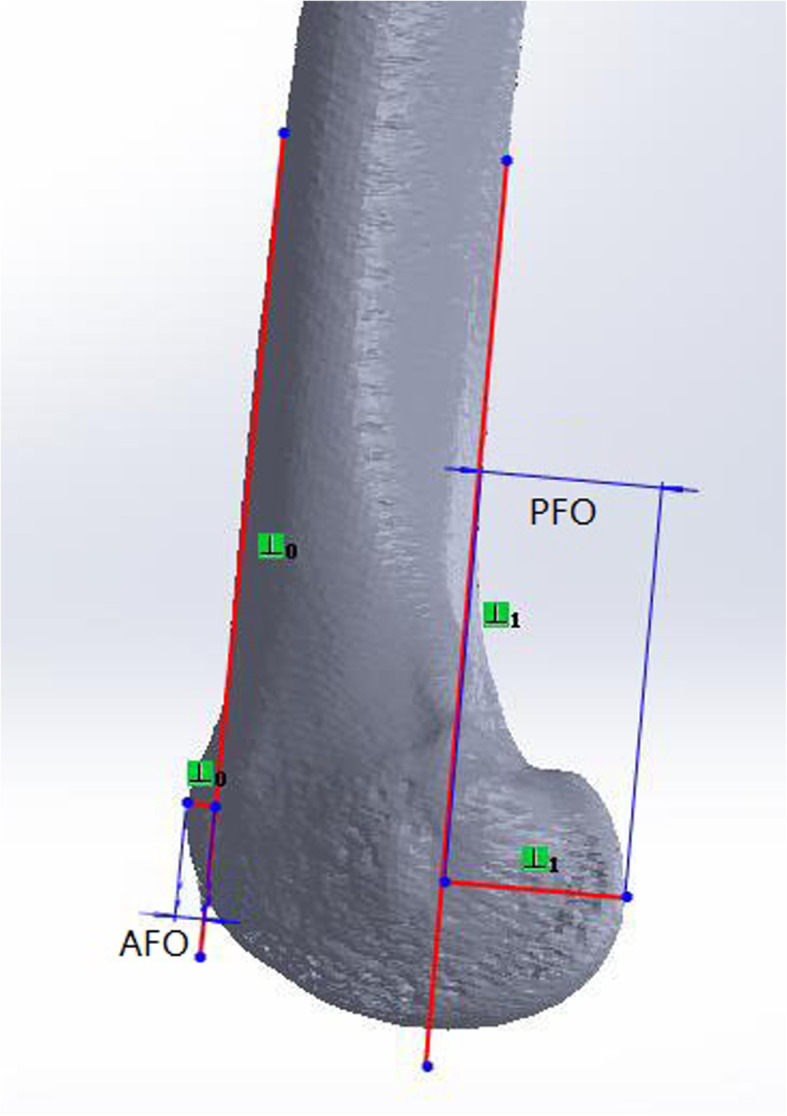
Anterior femoral offset (AFO) and posterior femoral offset (PFO)

In the lateral view of the femur, we measured the anterior femoral offset and the posterior femoral offset as described by Matz et al. and Voleti et al. respectively [3, 11] (Fig. 4):

1. Anterior femoral offset (AFO): the distance between the anterior edge of the femoral cortex and the anterior aspect of the anterior femoral condyle.
2. Posterior femoral offset (PFO): the distance between the posterior edge of the femoral cortex and the posterior aspect of the posterior femoral condyle.

**Fig. 5:**
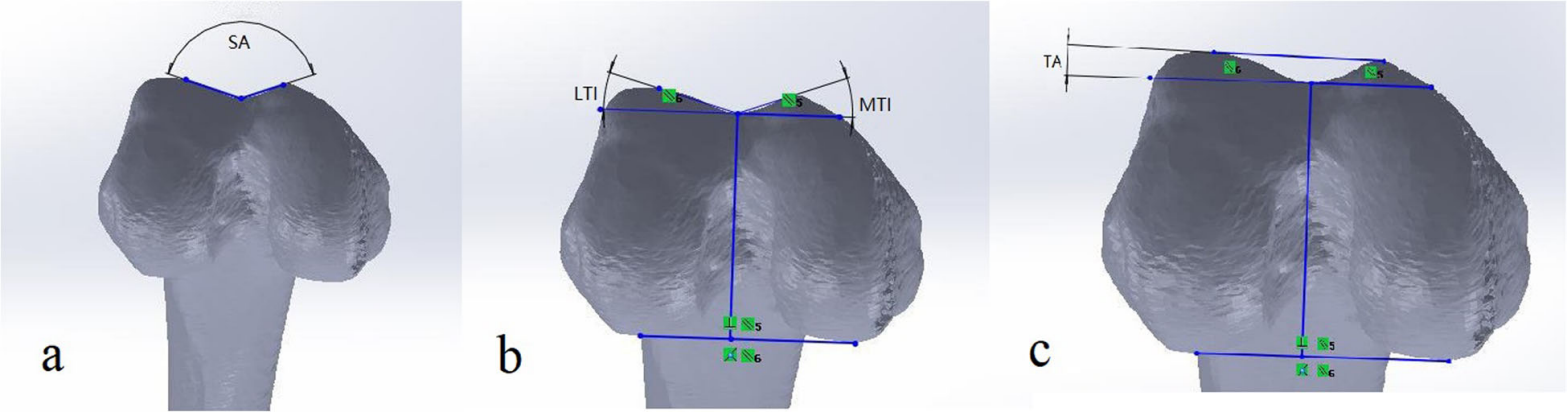
**a** Sulcus angle (SA); **b **lateral trochlear inclination (LTI), medial trochlear inclination (MTI); **(c)** trochlear angle (TA)

In the axial views simulated in 30° knee flexion of the femur, we measured the four following parameters as described by Stefanik et al. [34] (Fig. 5a-c):

1. Sulcus angle (SA): the angle between the two lines connecting the highest points of the medial and lateral condyles to the lowest point of the femoral sulcus.
2. Lateral and medial trochlear inclination (LTI, MTI): the angle between the PCL and the line connecting from the highest points of the lateral and medial condyles to the lowest point of the femoral sulcus, respectively. In addition, the SA, LTI, and MTI add up to 180°.
3. Trochlear angle (TA): the angle between the PCL and the line passing along the most anterior edge of the medial and lateral trochlear facets.

Two authors who had 12 and 8 years of experience with radiography took the measurements and repeated the measurements after 2 weeks.

### Statistical analysis

All statistical analyses were performed using R (version 3.5.1,R Foundation for Statistical Computing, Vienna, Austria), and *p* values less than 0.05 were considered significant. The intraclass correlation coefficient (ICC) was calculated to determine intrarater and interrater reliability, and the intrarater and interrater reliabilities were good to excellent (all ICCs > 0.85, Table 1).

**Table 1:** Reliability assessment

Variable	Interrater (ICC)	Intrarater (ICC)
PT(mm)	0.987	0.988
PFT(mm)	0.959	0.962
PW(mm)	0.935	0.974
PLFW(mm)	0.989	0.992
PLL(mm)	0.990	0.991
PAL(mm)	0.858	0.958
fML(mm)	0.967	0.971
fAP(mm)	0.956	0.959
tML(mm)	0.987	0.991
tAP(mm)	0.988	0.991
TTTG(mm)	0.923	0.956
AFO(mm)	0.959	0.973
PFO(mm)	0.988	0.988
SA(°)	0.976	0.984
LTI(°)	0.973	0.981
MTI(°)	0.973	0.982
TA(°)	0.979	0.979

*AFO *anterior femoral offset, *fAP *anteroposterior size of the femur, *fML *mediolateral size of the femur, *LTI *Lateral trochlear inclination, *MTI *medial trochlear inclination, *PAL *longitudinal length of the articulating surface of the patella, *PFO* posterior femoral offset, *PFT *patella facet thickness, *PLFW * patella lateral facet width, *PLL *longitudinal length of the whole patella, *PT *patella thickness, *PW *patella width, *SA* sulcus angle, *TA *trochlear angle, *tAP * anteroposterior size of the tibia, *tML *mediolateral size of the tibia *ICC *intraclass correlation coefficient

For normally distributed data, the two-sample Student’s t-test was performed to determine the significance of the difference between the sexes, otherwise, the Mann–Whitney U test was used. Next, multiple variable linear regression analysis was used to analyse the significant difference between the sexes again after controlling the bias from age, height, and weight. The Pearson correlation coefficients were calculated to explore the relationships among all parameters. We defined *r* < 0.3 as weak correlation, 0.3 < *r* < 0.8 as moderate correlation, and *r* > 0.8 as high correlation.

The least Absolute Shrinkage and Selection Operator(LASSO) regression models were constructed to predict the normal TT-TG distances. First, a boxplot was generated to determine the outliers from the distribution of TT-TG distances in men and women. Among women, the outliers were 15.02 mm and 14.80 mm, respectively, which were more than Q3 + 1.5*IQR. Among men, the outliers were 8.36 mm and 6.50 mm, respectively, which were less than Q1-1.5IQR (Additional file 2). With the purpose of predicting the TT-TG distances to meet most people, we removed the four outliers. The LASSO regression models were created using the “glmnet” package of R software. We selected the directly measured parameters above the moderate correlation coefficient with TT-TG distance to enter the model for coefficient progression. In addition, sex also entered the initial model. The LASSO regression introduces λ as a tuning parameter on the basis of linear regression, which controls the overall strength of the penalty. The greater the penalty is, the fewer parameters are retained in the model. Then the independent variable that has a strong influence on the dependent variable is selected and a relatively simplified model can be obtained. We used the mean squared error (MSE) as the selection criterion to describe the performance of the model. Tenfold cross-validation was automatically performed to calculate the λ value and MSE for a varying number of independent variables. We used λ at which the minimal MSE is achieved (lambda.min) and the largest λ at which the MSE is within one standard error of the minimal MSE (lambda.1se) to select the optimal model.

## Results

### Demographic data

After screening 102 participants, a total of 78 participants meeting the inclusion and exclusion criteria were included in the study. Women were younger (29 ± 5 years vs. 34 ± 3 years, *P* < 0.01), shorter (165 ± 3 cm vs. 177 ± 5 cm, *P* < 0.01), and weighed significantly less than men (57 ± 6 kg vs. 70 ± 9 kg, *P* < 0.01). The means and standard deviations for all parameters, as well as age, height, weight, and BMI, are shown in Table 2. Regarding the Wiberg classification as modified by Baumgartl and Ficat, types I, II and III accounted for 7.5 % (*n* = 3), 90 % (*n* = 36) and 2.5 % (*n* = 1) in men; and for 10.5 % (*n* = 4), 89.5 % (*n* = 34) and 0 (*n* = 0) in women, respectively. No other type was found. This indicates that most of the selected patellas were classified as type I and II, which were considered to be stable [35, 36].

**Table 2:** Demographic statistics and sex differences (*n* = 78 knees)

	men	women	overall	*p* value	*p*’ value
Age(y)	34 ± 3 (28 ~ 41)	29 ± 5 (21 ~ 40)	32 ± 5 (21 ~ 41)	< 0.01	
Height(cm)	177 ± 5 (168 ~ 185)	165 ± 3 (160 ~ 170)	171 ± 8 (160 ~ 185)	< 0.01	
Weight(kg)	70 ± 9 (58 ~ 95)	57 ± 6 (45 ~ 70)	64 ± 10 (46 ~ 90)	< 0.01	
BMI	22.37 ± 1.79 (19.60 ~ 27.72)	20.98 ± 2.18 (16.33 ~ 26.35)	21.70 ± 2.09 (16.88 ~ 26.38)	< 0.01	
PT(mm)	22.42 ± 1.57 (20.21 ~ 26.36)	19.80 ± 1.29 (17.33 ~ 22.85)	21.15 ± 1.94 (17.43 ~ 25.79)	< 0.01	0.188
PFT(mm)	11.77 ± 0.93 (10.33 ~ 13.54)	10.94 ± 0.96 (8.85 ~ 13.41)	11.36 ± 1.03 (9.13 ~ 13.46)	< 0.01	0.935
PW(mm)	46.94 ± 2.50 (41.73 ~ 53.55)	40.51 ± 2.34 (34.81 ~ 45.12)	43.81 ± 4.03 (36.72 ~ 52.22)	< 0.01	< 0.01
PLFW(mm)	27.18 ± 2.18 (22.52 ~ 33.06)	23.34 ± 1.59 (19.64 ~ 27.20)	25.31 ± 2.71 (20.41 ~ 31.26)	< 0.01	< 0.01
Wiberg index	0.58 ± 0.03 (0.51 ~ 0.69)	0.58 ± 0.03 (0.51 ~ 0.64)	0.58 ± 0.03 (0.51 ~ 0.65)	0.797	NA
PLL(mm)	45.21 ± 2.75 (38.88 ~ 51.36)	38.84 ± 2.64 (33.99 ~ 47.01)	42.11 ± 4.17 (34.39 ~ 51.08)	< 0.01	< 0.01
PAL(mm)	30.75 ± 2.43 (25.70 ~ 38.21)	26.82 ± 1.81 (22.89 ~ 31.65)	28.83 ± 2.91 (23.66 ~ 37.25)	< 0.01	0.089
fML(mm)	86.35 ± 3.99 (79.93 ~ 94.34)	74.14 ± 3.11 (67.51 ~ 82.44)	80.40 ± 7.10 (70.24 ~ 93.70)	< 0.01	< 0.01
fAP(mm)	66.78 ± 3.30 (60.66 ~ 74.32)	60.15 ± 2.81 (55.04 ~ 64.87)	63.54 ± 4.52 (55.57 ~ 72.52)	< 0.01	0.107
fML/fAP	1.29 ± 0.05 (1.23 ~ 1.49)	1.23 ± 0.05 (1.15 ~ 1.36)	1.26 ± 0.06 (1.15 ~ 1.37)	< 0.01	< 0.01
tML(mm)	80.46 ± 2.83 (75.83 ~ 87.70)	70.16 ± 2.52 (64.37 ~ 76.81)	75.44 ± 5.82 (66.31 ~ 86.48)	< 0.01	< 0.01
tAP(mm)	55.75 ± 3.13 (48.60 ~ 63.14)	48.72 ± 2.29 (44.27 ~ 53.75)	52.32 ± 4.47 (44.82 ~ 62.80)	< 0.01	< 0.01
TTTG(mm)	14.29 ± 2.11 (6.55 ~ 17.00)	12.92 ± 0.86 (11.10 ~ 15.02)	13.62 ± 1.76 (8.31 ~ 16.94)	< 0.01	0.090
AFO(mm)	6.37 ± 1.39 (3.90 ~ 8.96)	5.62 ± 1.37 (3.14 ~ 8.09)	6.01 ± 1.42 (3.36 ~ 8.60)	0.019	NA
PFO(mm)	26.77 ± 2.05 (22.77 ~ 31.54)	24.62 ± 1.78 (21.50 ~ 28.08)	25.73 ± 2.19 (21.77 ~ 30.12)	< 0.01	0.291
SA(°)	139.44 ± 4.95 (129.50 ~ 151.69)	138.20 ± 4.55 (125.36 ~ 148.80)	138.83 ± 4.77 (129.11 ~ 148.87)	0.254	NA
LTI(°)	20.92 ± 2.82 (15.57 ~ 27.93)	21.61 ± 3.71 (11.16 ~ 29.08)	21.25 ± 3.28 (14.93 ~ 28.48)	0.358	NA
MTI(°)	19.65 ± 3.58 (11.53 ~ 29.51)	20.20 ± 3.15 (13.31 ~ 27.73)	19.92 ± 3.37 (13.26 ~ 28.00)	0.473	NA
TA(°)	2.66 ± 1.97 (-1.87 ~ 6.34)	2.93 ± 2.28 (-2.86 ~ 7.12)	2.79 ± 2.11 (-1.92 ~ 6.44)	0.587	NA

Data are presented as the mean ± SD. The 2.5 and 97.5 percentiles are indicated in brackets

NA represents no linear relationship or not meeting the condition for linear regression analysis

*AFO *anterior femoral offset, *fAP *anteroposterior size of the femur, *fML *mediolateral size of the femur, *LTI *Lateral trochlear inclination, *MTI* medial trochlear inclination, *PAL *longitudinal length of the articulating surface of the patella, *PFO *posterior femoral offset, *PFT* patella facet thickness, *PLFW * patella lateral facet width, *PLL *longitudinal length of the whole patella, *PT*patella thickness, *PW *patella width, *SA* sulcus angle, *TA* trochlear angle, *tAP *anteroposterior size of the tibia, *tML *mediolateral size of the tibia

*p* values represent the significant differences between the sexes applying the two-sample t-test for independent samples

*p*’ values represent the significant differences between the sexes after controlling the bias from age, height, and weight using the multiple variable linear regression model

### Sex differences of patellofemoral measurements

When using a two-sample t-test for independent samples for sexual comparison, the six dimensions of the patella, mediolateral and anteroposterior sizes of the femur, femoral aspect ratio, mediolateral and anteroposterior sizes of the tibia, TT-TG distances, anterior femoral offset and posterior femoral offset of men were significantly larger than those of women, and the differences were statistically significant (all *p* values < 0.05). After controlling the bias from age, height, and weight, there were no significant differences in TT-TG distances, longitudinal length of the articular surface of the patella, or anterior-posterior dimensions including patella thickness, patella facet thickness, anteroposterior size of the femur, and posterior femoral offset between the sexes (all *p* values > 0.05). Additionally, other dimensions and femoral aspect ratios of men were still significantly larger than those of women (all *p* values < 0.05). No significant differences between the sexes were identified for the Wiberg index and the angles (all *p* values > 0.05). (Table 2)

### Pearson correlation coefficient analysis

**Fig. 6:**
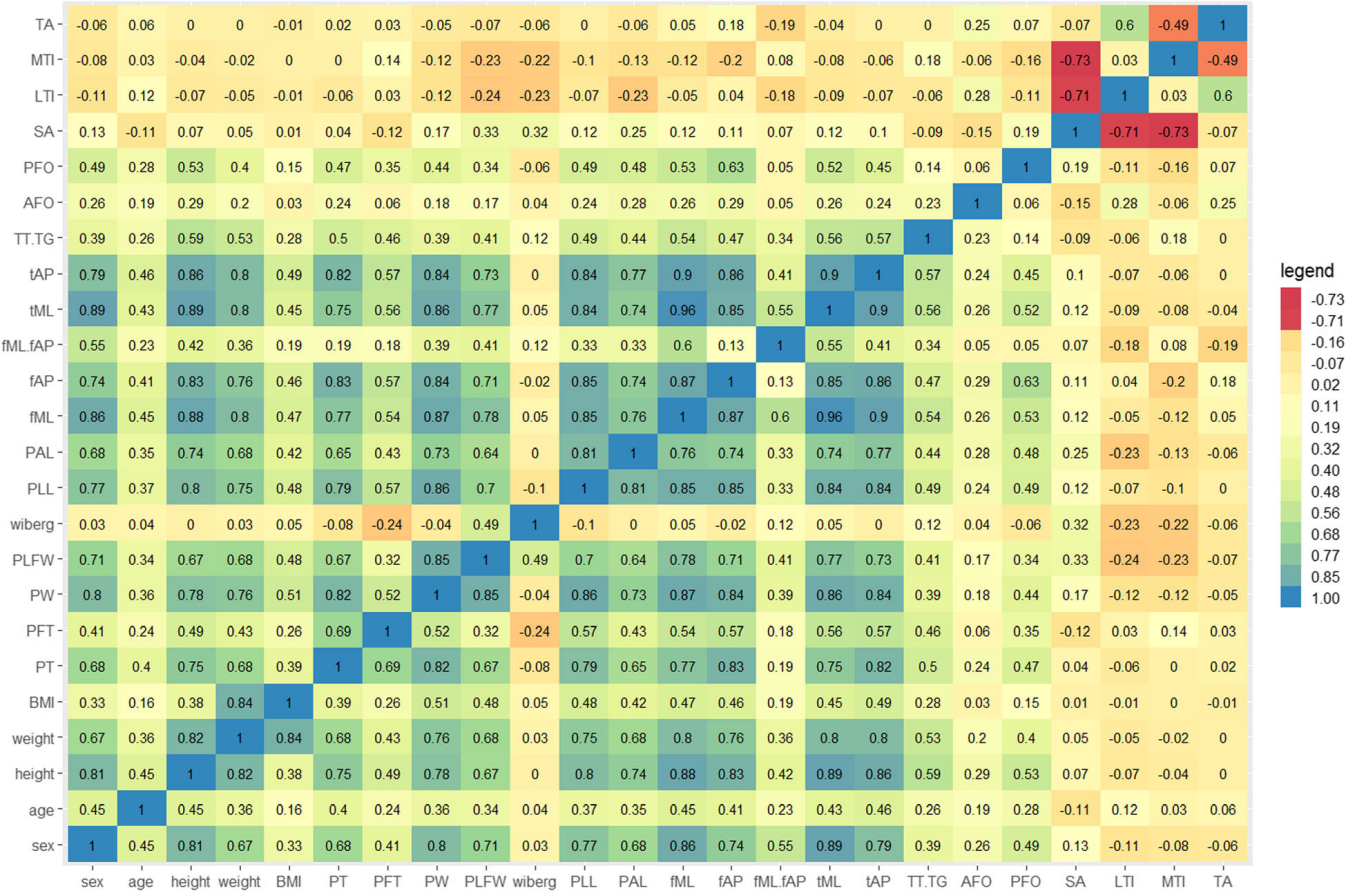
The Pearson correlation coefficients of all variables

1As shown in Fig. 6, the height, weight, dimensions of the patella, dimensions of the femur, dimensions of the tibia, and TT-TG distances were moderately-highly positively correlated with each other (r: 0.32 ~ 0.96, all *p* values < 0.05). In addition, the angles exhibited no or weak correlation with the dimensional parameters (*r* < 0.3), except that the sulcus angle was moderately correlated with patella lateral facet width (*r* = 0.33, *p* < 0.05). A medium correlation was found among angles (r: -0.49~-0.73, all *p* values < 0.05), except there was no correlation between lateral and medial trochlear inclination (*r* = 0.03, *p* = 0.79) or between the sulcus angle and trochlear angle (*r*=-0.07, *p* = 0.57).

Interestingly, the Pearson correlation coefficients between the anterior femoral offset and other parameters were consistently below 0.3, indicating no or weak relationship between anterior femoral offset and all other parameters. Similar results were observed for the sulcus angle and the Wiberg index.

### LASSO regression to predict normal TT-TG distance

**Fig. 7:**
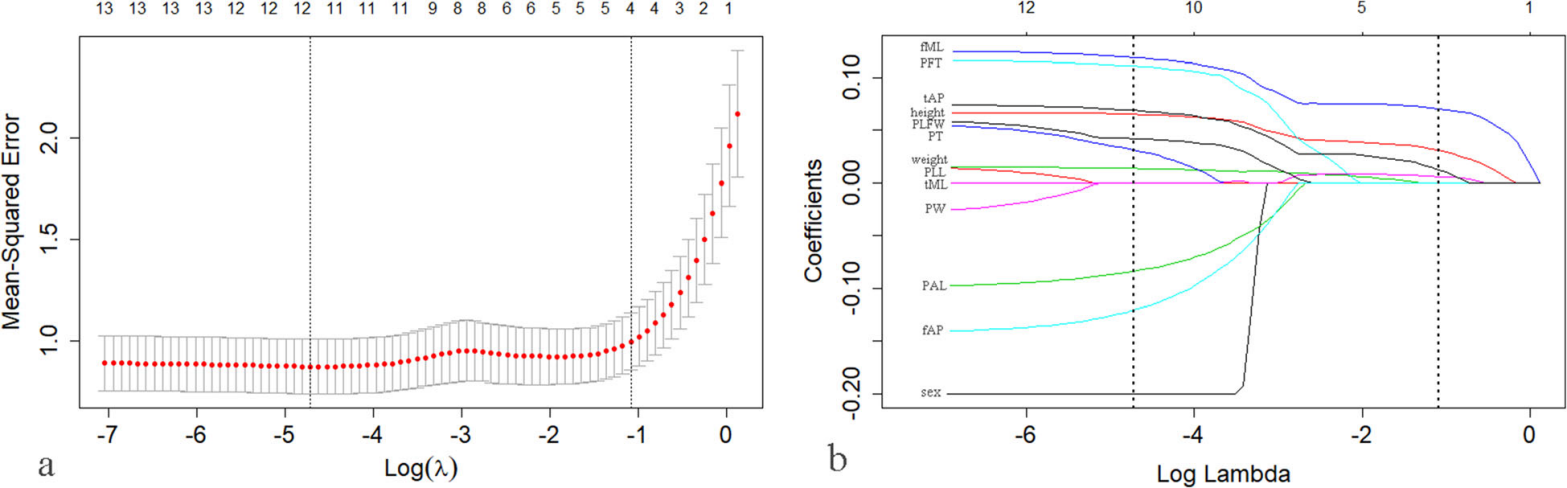
Coefficient progression with LASSO. **a** As the parameters shrink, the mean-squared error flattens at first and increases rapidly after four parameters are retained. **b** The change in the coefficient of each parameter as the parameters shrink. The first dotted line means that we used the λ at which the minimal mean squared error (MSE) is achieved (lambda.min) to select the optional model. The second dotted line means that we used the largest λ at which the MSE is within one standard error of the minimal MSE (lambda.1se) to select the optional model. The latter was selected as the final model in this study

A LASSO regression model was constructed to analyse the prediction of the normal TT-TG distances. Thirteen parameters were selected into the model. Coefficient progression is shown in Fig. 7. Taking lambda.min as a reference, 11 parameters were included and only PW and PLL were excluded (R^2^ = 0.7052, *p* < 0.01), which was not convenient to calculate. Thus we took lambda.1se as a reference, and height, fML, tML, and tAP were included in the final model (R^2^ = 0.5612, *p* < 0.01). The formula is defined as:

$$ ``\mathrm{TT}-\mathrm{TGdistance}=\mathrm{height}\ast 0.029+\mathrm{fML}\ast 0.069+\mathrm{tML}\ast 0.005+\mathrm{tAP}\ast 0.010+2.307" $$

The height is expressed in cm, while the fML, tML, and tAP are expressed in mm. In this study, only data with heights ranging from 160 to 185 cm, fML ranging from 67.51 to 94.36 mm, tML ranging from 64.37 to 87.73 mm, and tAP ranging from 44.27 to 63.15 mm were included in the LASSO regression model. Thus, this formula might not be available to populations beyond that range. Sex was excluded from this model, which indicated that sex had little influence on the predictive performance of the model. Taken together, on the premise of ensuring high model quality, we reduced the parameters to the minimum and established a formula that is convenient to calculate.

## Discussion

In the past few decades, the development of TKA has been considerable, but there is a high rate of dissatisfaction, often due to patellofemoral pathologies. In addition to surgical techniques, inappropriate prosthetic design often results in mismatches of the patellofemoral joints [37, 38]. In this study, we performed comprehensive measurements of patellofemoral joints as a whole and found dimensional and shape differences between the sexes. Next, we exploratively found that the anterior femoral offset, sulcus angle, and Wiberg index all varied greatly among individuals. Finally, we found that the TT-TG distances were moderately correlated with the height, weight, and dimensional parameters. Applying the LASSO regression model, we used four parameters to predict the normal values of the TT-TG distances, namely height, fML, tML, and tAP, to achieve the best accuracy and convenience.

Many studies have reported measurements of patella thickness. The comparisons of patellar thickness with those measured in other studies are shown in Table 3. The results indicated that the patella thickness of Chinese individuals tended to be smaller than that of the Whites, comparable to that of Koreans and greater than that of Indians [13, 14, 31, 39–41]. This finding is consistent with the results from previous studies [12, 13, 31]. The re-establishment of original thickness and adequate residual bone thickness is considered a key surgery guideline in TKA [42]. However, due to the mismatch of the patellar implants, the surgeons had to choose between the re-establishment of original thickness and adequate residual bone thickness. By choosing the former, the low residual bone thickness likely causes fracture and instability; by choosing the latter, the increased thickness of the patella causes overstuffing of the patellofemoral joint and leads to anterior knee pain [43, 44]. Although several studies have shown that adverse clinical outcomes were unlikely to occur if the overall and residual bone thickness of the patella was maintained in a reasonable range (postoperative thickness within 3 mm of the original thickness of the patella, and residual thickness between 10 and 15 mm), the changes in the patella might affect the patellofemoral contact pressures, thus leading to complications of the patellofemoral joint [12]. Therefore, patellar prostheses with more available choices should be designed according to patellar characteristics in Chinese population.

**Table 3:** Comparison of patella thickness (mm) with the data from the literature

Population studied	men	women	study
Whites	23.9	21.8	Baldwin et al.
Whites	26.1	22.6	Chmell et al.
Whites	25.3	22.5	Hitt et al.
Whites	24.9	21.0	Rooney et al.
Korean	22.7	20.4	Yoo et al.
Indian	20.3	16.2	Muhamed et al.
Chinese	22.42	19.80	Present study

We explained many sex differences from this study. The results showed that the dimensional parameters of men were generally larger than those of women, which was consistent with previous studies [7, 14]. In terms of shape, after controlling for the bias from age, height, and weight, there were no significant differences in anterior-posterior dimensions, including patella thickness, patella facet thickness, the anteroposterior size of the femur, and posterior femoral offset, between the sexes, while other dimensions and the femoral aspect ratio of men were still significantly larger than those of women. This indicated that the patella and femur of women were thicker than those of men in the anterior-posterior direction for the same medial-lateral dimensions, which was consistent with the relatively small femoral aspect ratio in women. Therefore, the shape of the distal femur of men was more “flatter” than that of women, while women had a “narrower” distal femur than men. These results were comparable to those reported in previous studies [7, 9, 15, 16]. We found that the Wiberg index and the shape of the trochlea exhibited no dimorphisms between the sexes. Gillespie et al. reported that no significant difference between the sexes was found in the medial and lateral flanges, which was similar to our results [16]. Based on these features, sex-specific prostheses should be designed in consideration of sex characteristics. However, an increasing number of studies have focused on not only sex differences but also individual differences [8, 15]. Taking this issue into account, we explored the correlation coefficient between all parameters.

This study found that anterior femoral offset, sulcus angle, and the Wiberg index, as the primary description of the patellofemoral shape and thickness, all exhibited no or weak relationship with other parameters, which indicated that these three parameters varied greatly regardless of the sizes and shape of the knees. Further analysis of the results indicated that these three parameters varied greatly among individuals, which might need to be considered in the design of joint prostheses. To avoid overstuffing and notching of the patellofemoral compartment, AFO should be treated appropriately. Matz et al. reported that the probability of changes in AFO after TKA was 40 % compared with that before TKA [3]. Although some previous studies showed no significant differences between AFO restoration and clinical outcomes, there was a trend towards improved outcomes [3, 45]. Other studies showed that if the AFO increased after TKA and there was a risk of overstuffing due to the mismatch of the prosthesis, the pressure of the patellofemoral joint would increase, and then, there would be complications such as anterior knee pain and decreased knee motion [46]. Taking these issues into account, an increasing number of studies have analysed the shape and variance of the distal femur. Lonner and Gillespie et al. indicated that the overall variability of the anatomy of the distal femur should be taken into account but not sex differences [16, 17]. According to the individual differences, Everhart et al. proposed a binary classification system to describe the shape of the distal femur and five binary categories were selected based on the aspect ratio, trochlear width, trochlear tilt, the ratio of medial and lateral trochlear width, and trochlear groove angle [47]. In addition, Varadarajan et al. reported that the laterally oriented proximal part and medially oriented distal part formed the intact trochlear groove, and there was a turning point to distinguish these two parts [48]. Moreover, Chen et al. proposed a quaternary system based on the position of the turning point [49]. Due to the great individual variance of the distal femur, more studies on different shapes of the femoral components should be focused, and prosthetic implants with greater varieties in sizes and shapes of anterior femoral condyles need to be designed.

The TT-TG distance had a significant positive correlation with the tubercle sulcus angle (TSA) and Q-angle and was considered to be objective and reliable in the quantification of extensor mechanism malalignment and patellar instability [25, 28]. In previous studies, the measurement of the TT-TG distance was mainly used in image overlapping technology based on CT and MRI. However, several studies have reported the inaccuracy of the current measurement [25, 50], and we found that mild adduction or abduction of the lower extremities resulted in a greater change in this value. In this study, we took this issue into account, and used the online reslice of the Mimics software to standardize the selection of images, so that the collected transverse picture was as perpendicular to the vertical axis of the lower limb as possible, which greatly ensured the accuracy of measurement.

This study reported the average CT-based TT-TG distance to be 13.62 ± 1.76 mm. The average TT-TG distance from the research of Hernigou et al. was 13 mm, which was measured based on CT data and was similar to our results [28]. Tse et al. showed by MRI that the average TT-TG distance was 10.1 mm in Chinese individuals [51]. In a study conducted in New Zealand, Pandit et al. reported the average MRI-based values to be 9.91 mm for men and 10.04 mm for women [21]. Hinckel et al. reported that the MRI-based TT-TG distance was 3.1–3.6 mm smaller than the CT-based TT-TG distance, which explained the inconsistency of the above results [52]. At present, an increasing number of studies have recognized the limitation of the absolute threshold of the TT-TG distance. Although 20 mm was the main diagnostic threshold for surgical application, there are some disputes about its value. Franciozi et al. reported that tibial tubercle osteotomy combined with medial patellofemoral ligament reconstruction (MPFLR) resulted in better outcomes than MPFLR alone in the treatment of recurrent patellar instabilities in patients with a TT-TG distance of 17 to 20 mm [26]. Graf et al. reported the inaccuracy of surgical intervention and demonstrated the need for combining the TSA and TT-TG distances to avoid overcorrection during medial tibial tubercle osteotomy [25]. Our results reported that the TT-TG distance had a positive correlation with height and knee size, which was comparable to other studies [28, 53, 54]. Moreover, several studies have described that the application of TT-TG indices (the ratio of the TT-TG distance to the tibial maximal mediolateral axis) obtained more reliable and standardized results, but the results needed to be further confirmed [27, 54]. Hernigou et al. used fML and tML to establish normal TT-TG distances in Belgium. However, they also raised doubts about whether the two parameters were applied as the best predictors [28]. Taking these questions into account, the present study applied the LASSO regression model to analyse the best predictors of normal TT-TG distances. LASSO regression is a machine learning method that can shrink the coefficients of variables that do not contribute information to the model to zero and is well suited to feature selection for high-dimensional data [55]. Using this method, we obtained four parameters to predict the normal TT-TG distance, namely, height, fML, tML and tAP, to achieve the best accuracy and convenience. The prediction formula obtained by us might provide a more accurate reference for the clinical determination of patellar instability, rather than the absolute values or TT-TG indices, which needs further study to validate the results. Additionally, as Hernigou et al. described, they predicted the restored location of the tibial tuberosity using the mediolateral distances of the femur and the tibia when performing medial transfer of the tibial tuberosity. The formula could play a guiding role in the accurate restored localization of the tibial tubercle during tibial tubercle osteotomy, but this needs to be validated by further research.

Limitations of the present study include the relatively small sample size. We are continuing to recruit more participants to increase the validity of the anatomical data. Another limitation of the present study was that the formula for predicting TT-TG distance has not been clinically verified, and more studies on the clinical effectiveness of the formula need to be performed.

## Conclusions

Normative data of patellofemoral morphology were provided for the Chinese population. In summary, the dimensional indexes of men were generally larger than those of women. In terms of shape, the patella and femur of women were thicker than those of men in the anterior-posterior direction for the same medial-lateral dimensions. Moreover, the anterior femoral offset, sulcus angle, and Wiberg index all varied greatly among individuals. More attention should be devoted to not only sex differences but also individual differences. In addition, using LASSO regression, we obtained four parameters to predict normal TT-TG distances, namely, height, mediolateral size of the femur, and mediolateral and anteroposterior sizes of the tibia, to achieve the best accuracy and convenience. This study provided a reference for prosthetic design and a new method to predict TT-TG distances accurately.

## Supplementary Information

**Additional file 1.**

**Additional file 2.**

## Data Availability

The dataset supporting the conclusions of this article is included within the article and its additional file (Additional file 1).

## Abbreviations

AFO: Anterior femoral offset
CT: Computed tomography
fAP: Anteroposterior size of the femur
fML: Mediolateral size of the femur
ICC: Intraclass correlation coefficient
lambda.min: The λ at which the minimal MSE is achieved
lambda.1se: The largest λ at which the MSE is within one standard error of the minimal MSE
LASSO: Least Absolute Shrinkage and Selection Operator
LTI: Lateral trochlear inclination
MSE: Mean squared error
MTI: Medial trochlear inclination
PAL: Longitudinal length of the articulating surface of the patella
PCL: Posterior condylar line
PFO: Posterior femoral offset
PFT: Patella facet thickness
PLFW: Patella lateral facet width
PLL: Longitudinal length of the whole patella
PT: Patella thickness
PW: Patella width
SA: Sulcus angle
TA: Trochlear angle
tAP: Anteroposterior size of the tibia
TKA: Total knee arthroplasty
tML: Mediolateral size of the tibia
TSA: Tubercle sulcus angle
TT-TG distance: The tibial tubercle-trochlear groove distance
