# Supplementary figures and images for: The patellofemoral morphology and the normal predicted value of tibial tuberosity-trochlear groove distance in the Chinese population
Source: BMC Musculoskelet Disord. 2021 Jun 23;22:575. doi: 10.1186/s12891-021-04454-8 (PMC8223279; doi:10.1186/s12891-021-04454-8)

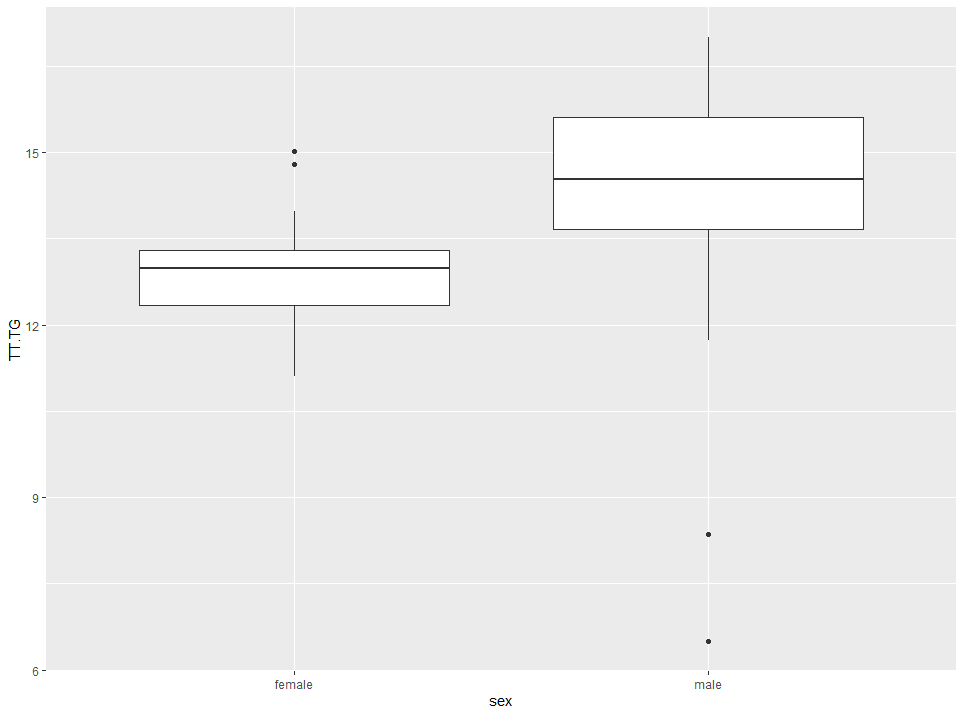

Supplement: Supplementary file 2 — Additional file 2. [file 12891_2021_4454_MOESM2_ESM.tiff]
